# Bone Concentration of Ampicillin/Sulbactam: A Pilot Study in Patients with Osteonecrosis of the Jaw

**DOI:** 10.3390/ijerph192214917

**Published:** 2022-11-13

**Authors:** Anton Straub, Maximilian Stapf, Markus Fischer, Andreas Vollmer, Christian Linz, Thiên-Trí Lâm, Alexander Kübler, Roman C. Brands, Oliver Scherf-Clavel, Stefan Hartmann

**Affiliations:** 1 Department of Oral and Maxillofacial Plastic Surgery, University Hospital Wuerzburg, Pleicherwall 2, 97070 Wuerzburg, Germany; 2 Institute for Pharmacy and Food Chemistry, University of Wuerzburg, Am Hubland, 97074 Wuerzburg, Germany; 3 Institute for Hygiene and Microbiology, University of Wuerzburg, Josef-Schneider-Str. 2/E1, 97080 Wuerzburg, Germany

**Keywords:** osteonecrosis of the jaw, ARONJ, MRONJ, ONJ, osteoradionecrosis, antibiotic bone concentration, jaw bone, beta-lactam, ampicillin

## Abstract

Osteonecrosis of the jaw (ONJ) occurs typically after irradiation of the head and neck area or after the intake of antiresorptive agents. Both interventions can lead to compromised bone perfusion and can ultimately result in infection and necrosis. Treatment usually consists of surgical necrosectomy and prolonged antibiotic therapy, usually through beta-lactams such as ampicillin/sulbactam. The poor blood supply in particular raises the question as to whether this form of antibiosis can achieve sufficient concentrations in the bone. Therefore, we investigated the antibiotic concentration in plasma and bone samples in a prospective study. Bone samples were collected from the necrosis core and in the vital surrounding bone. The measured concentrations in plasma for ampicillin and sulbactam were 126.3 ± 77.6 and 60.2 ± 35.0 µg/mL, respectively. In vital bone and necrotic bone samples, the ampicillin/sulbactam concentrations were 6.3 ± 7.8/1.8 ± 2.0 µg/g and 4.9 ± 7.0/1.7 ± 1.7 µg/g, respectively. These concentrations are substantially lower than described in the literature. However, the concentration seems sufficient to kill most bacteria, such as *Streptococci* and *Staphylococci*, which are mostly present in the biofilm of ONJ. We, therefore, conclude that intravenous administration of ampicillin/sulbactam remains a valuable treatment in the therapy of ONJ. Nevertheless, increasing resistance of *Escherichia coli* towards beta-lactam antibiotics have been reported and should be considered.

## 1. Introduction

Osteonecrosis of the jaw (ONJ) usually occurs after radiation therapy to the head and neck area or after the intake of antiresorptive drugs, such as bisphosphonates or denosumab. ONJs are therefore classified as either osteoradionecrosis of the jaw (ORN) or antiresorptive-agent-related necrosis of the jaw (MRONJ) [1,2,3]. Antiresorptive agents are mainly administered in patients with osteoporosis, bone metastases, multiple myeloma, leukemia, or fibrous dysplasia. Diagnosis of MRONJ is only possible when all the following three conditions are met: (1) current or previous treatment with antiresorptive drugs, (2) exposed bone in the maxillofacial region that persists for more than eight weeks, and (3) no history of radiation therapy to the jaws or metastatic disease of the jaws [1]. In contrast, ORN is diagnosed when exposed bone in the maxillofacial region is detected after irradiation of the head and neck region has been performed. Radiation therapy of the head and neck area is usually applied either as primary or adjuvant therapy of head and neck squamous cell carcinoma [4].

Irradiation damages the jaw bones in a different way than the intake of antiresorptive drugs; however, there are similarities in the development of necrosis from the pre-damaged bone. The pathophysiology of osteonecrosis is complex, not completely understood to date, and differs in both diseases. There is decreased blood supply to the bone, which in the case of MRONJ is caused by inhibition of bone remodeling, increasing bone density. This is evoked by the inhibitory effect on osteoclasts and the stimulation of osteoblasts. Furthermore, bisphosphonates have a direct toxic effect on soft tissue and bone and suppress angiogenesis [1,5,6,7].

On the other hand, in the case of ORN, there is direct damage to the bone substance and hyalinization, in addition to thrombosis of the supplying blood vessels. Recent research further postulated that deregulated fibroblast activity leads to a fibroatrophic environment, which ends up in a hypocellular and hypovascularized bone [2,8,9].

Both result in a reduced remodeling of the bone. Bacterial colonization leads to infection and necrosis when the mucosal integrity is injured, for example, by tooth extraction, micro-damage, or prosthesis pressure points. Infection and bacterial colonization of the affected bone are crucial steps in the development of ONJ [8,10].

There are conservative and surgical treatment options. Conservative treatment options are useful at all stages and can stabilize and cure MRONJ, especially in the early stages. Furthermore, it is an important treatment option when surgery is not possible (for example comorbidities) [1,11]. Nevertheless, most patients undergo surgery because of the lower success rate, prolonged therapy, and the progression of ONJ when only conservative therapy is performed [12,13]. Surgical treatment involves complete necrosectomy of the bone and mucosal closure or other reconstructive surgery, such as microvascular transplants. However, surgery does come with the risk of complications, for example, dehiscence, infections, and re-exposed bone, due to the compromised blood supply of the bone [12,14,15]. To reduce the risk of these complications occurring, the current guidelines recommend prolonged antibiotic therapy from surgery until stitch removal either with ampicillin/sulbactam or clindamycin [1]. However, it is unclear if the antibiotic load in the necrotic and surrounding bone is sufficient to inhibit bacterial growth locally, which is rather crucial in prophylaxis and even more so in the treatment of this disease. Only sufficient antibiotic loads can prevent bacterial colonization and ONJ, for example after tooth extraction. There are a number of interesting approaches to solving this problem, for example by using platelet-rich fibrin (PRF) to apply antibiotics locally [16].

In 2005, Heibel et al. investigated the bone concentration of ampicillin/sulbactam after neo-adjuvant radiation therapy of the mandible. The study revealed antibiotic concentrations three to four times lower in patients having undergone radiation therapy compared to patients without radiation therapy. Nevertheless, the concentration of ampicillin/sulbactam in the irradiated bone was higher than the minimal inhibitory concentration (MIC) of *Streptococci* and *Staphylococci* [17]. Therefore, it was concluded that antibiotic therapy remains an effective method to prevent ONJ.

However, the pathomechanism of ONJ suggests that systemic antibiotic administration may be effective in prevention, but less so or even ineffective in the treatment of the disease. We thus hypothesized that the antibiotics do not reach the region of interest because of the compromised blood supply to the necrotic bone. Furthermore, the local antibiotic concentration may potentially be high enough to deal with *Streptococci* and *Staphylococci*, but not with *Escherichia coli*, which is known to have a higher MIC [18].

In this study, we investigated the concentration of ampicillin and sulbactam in vital and necrotic bone samples in patients suffering from ONJ caused either by radiation or medication. Furthermore, we measured the ampicillin/sulbactam concentration in the plasma of these patients. To the best of our knowledge, this is the first study investigating the antibiotic concentration in bone samples of the jaw taken from patients suffering from ONJ.

## 2. Materials and Methods

**Figure 1:**
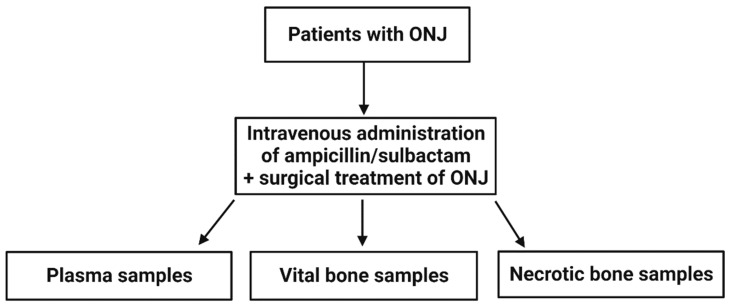
Flowchart—all consenting patients with osteonecrosis of the jaw (ONJ) either after radiation of the head and neck area or after the intake of antiresorptive agents were included in the study when inclusion criteria matched. Where possible, a plasma sample as well as necrotic and vital bone sample were collected from each patient.

We initiated a prospective study from October 2020 to November 2021, in which we investigated the concentration of ampicillin/sulbactam in vital and necrotic bone samples biopsied from patients suffering from ONJ (see Figure 1).

Inclusion criteria were a diagnosis of ONJ either after intake of antiresorptive drugs or after radiation therapy to the head and neck area, as well as intravenous antibiotic therapy with ampicillin/sulbactam, and surgical treatment of the ONJ. Furthermore, an age of at least 18 years was also set as inclusion criterion.

Patients were excluded from participating if allergic to penicillin, if the cause of their ONJ was anything other than MRONJ or ORN (e.g., osteomyelitis), or when they failed to comply with study protocols after being included (e.g., neither plasma nor vital or necrotic bone samples could be obtained).

The Ethics Committee of the University of Würzburg approved all the protocols implemented in this study (IRB approval number: 51/20-me and 143/20-me). Written informed consent was obtained from all participants prior to inclusion.

### 2.1. Antibiotic Therapy

Participants were admitted to hospital one day prior to surgical intervention and antibiotic therapy comprising ampicillin/sulbactam (Unacid^®^, Pfizer Pharma GmbH, Berlin, Germany) at a dose of 2 g/1 g every eight hours was started on the day of admission. During surgery, 2 g/1 g of ampicillin/sulbactam was again administered as perioperative prophylaxis. According to this protocol, every patient received at least three doses of ampicillin/sulbactam, at a ratio of 2 g/1 g, prior to surgery and one additional dose intraoperatively.

### 2.2. Plasma

Blood sampling to determine plasma concentrations was performed ten minutes after intravenous administration of 2 g/1 g ampicillin/sulbactam intraoperatively. Blood for the plasma sample was collected via venepuncture in a 1.6 mL EDTA tube (S-Monovette, Sarstedt, Sarstedt-Straße 1, 51588 Nümbrecht, Germany) and centrifuged (4900 rpm for ten minutes and 4 °C). Four aliquots of 100 µL were frozen at −80 °C. The concentrations of ampicillin and sulbactam were measured at the Institute of Pharmacy of the University of Würzburg (see Section 2.2.1 below).

#### 2.2.1. Quantification of Ampicillin/Sulbactam Levels in Plasma

A specific liquid chromatography–tandem mass spectrometry (LC-MS/MS) method was developed and validated according to the European Medicines Agency (EMA) guidelines on bioanalytical method validation [19]. The final method for plasma and for jawbone matrix met the requirements of the authority (EMA) in terms of sensitivity, linearity, selectivity, carryover, within-run and between-run accuracy and precision, matrix effect, and extraction recovery. Blank EDTA plasma was used to prepare calibration and quality control samples. Samples were monitored through electrospray ionization in the multiple-reaction-monitoring mode. Ampicillin was measured in the positive-ion mode and sulbactam in the negative-ion mode (MRM transitions used for quantification: *m*/*z* 350.0 → 106.0 for ampicillin; *m*/*z* 355.2 → 111.0 for the corresponding internal standard ampicillin-d5; *m*/*z* 231.9 → 63.8 for sulbactam; *m*/*z* 236.8 → 63.7 for internal standard sulbactam-d5). Protein precipitation using acetonitrile was applied in the sample preparation of plasma. The lower limit of quantification (LLOQ) of the plasma method was 2 µg/mL for both ampicillin and sulbactam.

### 2.3. Vital and Necrotic Bone Samples

**Figure 2:**
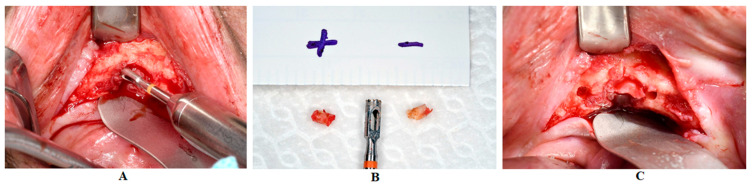
Surgical site of a patient suffering from ORN. (**A**) Illustrates the collection of a necrotic bone sample with a rotating trepan drill. (**B**) Vital bone sample (+) and necrotic bone sample (−). (**C**) Surgical site after the collection of two bone samples (vital and necrotic).

Necrotic bone samples were obtained from the center of the ONJ either with forceps or with rotating instruments. In the same way, the vital bone samples were taken from the marginal area not affected by ONJ. Clinical parameters such as bone bleeding and visual appearance helped to identify vital bone areas. The minimum diameter of the bone samples was 3 mm (see Figure 2).

**Figure 3:**
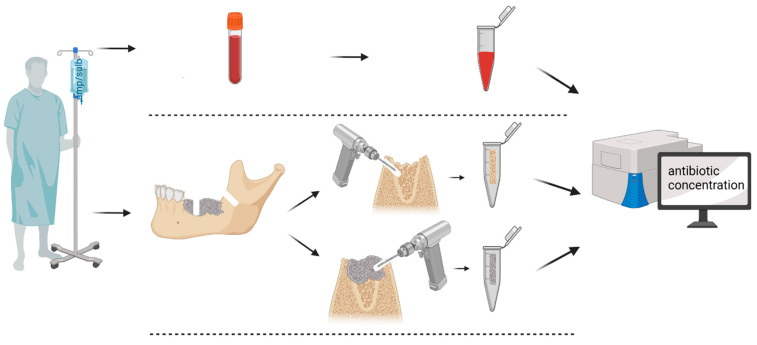
Ampicillin/sulbactam was administered to each patient intravenously on admission to hospital. This routine was started one day before surgery. Ampicillin/sulbactam was again administered as perioperative prophylaxis ten minutes before the plasma blood sample was taken. Bone samples were normally obtained within 60 min after antibiotic injection, depending on the surgical process. The necrotic sample was taken from the center of the ONJ, and the vital sample from the surrounding healthy bone tissue. The samples were further processed as described above.

The samples were stored immediately at −80°C in their untreated state until undergoing further processing in the Institute of Pharmacy of the University of Würzburg (see Section 2.3.1 and Figure 3).

#### 2.3.1. Quantification of Ampicillin/Sulbactam Levels in Vital and Necrotic Bone Samples

Cleaned vital and necrotic bone samples were pulverized under liquid nitrogen using a cryogenic mill (SPEX CertiPrep Freezer/Mill 6850). The pulverized samples were further processed using protein precipitation. Here, 80% methanol was used as the precipitating agent. The LLOQ of the bone method was 0.15 µg/g for ampicillin and 0.25 µg/g for sulbactam. Monitoring of the bone samples was analogous to plasma as described in the previous section. Calibration and quality control samples were generated by spiking defined amounts of blank porcine jawbone powder with aqueous solutions of the analytes containing ampicillin and sulbactam in the appropriate concentrations.

### 2.4. Statistics

Descriptive statistical analyses, the Wilcoxon signed-rank test, and Spearman‘s rho were performed with GraphPad Prism, version 9 (GraphPad Software, San Diego, CA, USA).

## 3. Results

### 3.1. Descriptive Statistics

We enrolled 21 patients in this study, collecting 21 necrotic and 13 vital bone samples in total. We were able to obtain plasma from all but two patients (*n* = 19). The main reason we only obtained necrotic but not vital bone samples in eight patients was the surgeon’s decision not to enlarge the surgical site. The mean age of patients was 69 years with a slight predominance of males (52.3%). All participants suffered from ONJ, either after radiation therapy (ORN) or following the intake of antiresorptive drugs (MRONJ) (Table 1).

**Figure 4:**
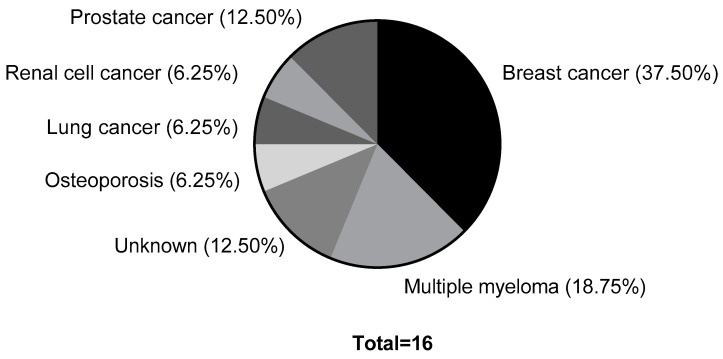
Etiology of MRONJ: six patients with breast cancer, three patients with multiple myeloma, two patients with prostate carcinoma, and one case each of renal cell carcinoma, lung cancer, and osteoporosis. In two cases, the reason for medication intake is unknown.

Regarding the etiology of MRONJ (*n* = 16), osseous metastatic breast carcinoma was present in 37.5% of the cases (*n* = 6), multiple myeloma in 18.8% of the cases (*n* = 3), osseous metastatic prostate carcinoma in 12.5% of the cases (*n* = 2), osseous metastatic renal cell carcinoma, as well as lung cancer and osteoporosis in 6.2% of the cases each (each *n* = 1). The etiology was unknown in two cases (see Figure 4).

All patients with ORN (*n* = 5) underwent primary or adjuvant radio(chemo)therapy after oral squamous cell cancer. The mean time interval between irradiation and occurrence of ORN was 7.5 ± 13.1 years.

### 3.2. Ampicillin/Sulbactam Concentration in Plasma

The ampicillin/sulbactam concentration in plasma was determined in 19 patients and revealed a mean concentration of ampicillin of 126.3 µg/mL (SD ± 77.6) and a mean concentration of sulbactam of 60.2 µg/mL (SD ± 35.0). The 95% confidence interval was 88.8–163.8 for ampicillin and 43.3–71.1 for sulbactam (see Table 2).

### 3.3. Ampicillin/Sulbactam Concentrations in Vital and Necrotic Bone Samples

**Figure 5:**
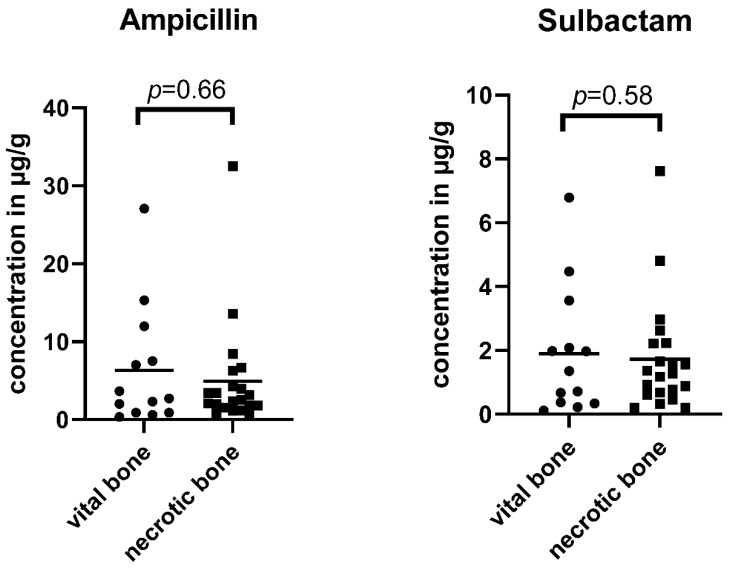
Ampicillin and sulbactam concentrations in vital and necrotic bone samples. For ampicillin, a mean concentration of 6.3 µg/g (SD ± 7.8 µg/g) in vital and 4.9 µg/g (SD ± 7.0 µg/g) in necrotic bone samples was detected. The values for sulbactam were 1.9 µg/g (SD ± 2.0 µg/g) in vital and 1.7 µg/g (SD ± 1.7 µg/g) in necrotic bone samples.

The mean ampicillin and sulbactam concentrations in vital bone samples (*n* = 13) were 6.3 µg/g (SD ± 7.8 µg/g) and 1.9 µg/g (SD ± 2.0 µg/g), respectively. The 95% confidence interval for ampicillin was 1.6–11.0 and 0.7–3.1 for sulbactam (see Table 3 and Figure 5).

The mean ampicillin and sulbactam concentrations in necrotic bone samples (*n* = 21) were 4.9 µg/g (SD ± 7.0 µg/g) and 1.7 µg/g (SD ± 1.7 µg/g), respectively. The 95% confidence interval for ampicillin was 1.7–8.1 and 0.9–2.5 for sulbactam (see Table 4 and Figure 5).

The minimum values measured for sulbactam were 0.1 µg/g in vital bone and 0.2 µg/g in necrotic bone. These values were below the LLOQ and therefore not validated. The lowest validated values for sulbactam in vital and necrotic bone are portrayed in Table 3 and Table 4.

As subgroup analyses (MRONJ versus ORN and maxilla versus mandible) were statistically not sufficient, we provide the mean concentration of ampicillin/sulbactam in these cases as Supplementary Materials (Tables S1 and S2).

### 3.4. Differences between Vital and Necrotic Bone Concentrations

The Wilcoxon signed-rank test revealed no significant difference (α < 0.05) between vital and necrotic bone samples, neither for ampicillin (*p* = 0.52) nor for sulbactam (*p* = 0.79).

A positive correlation between the plasma concentration of ampicillin and sulbactam and vital bone samples was detected with Spearman’s rho test (ampicillin: ρ = 0.92/*p* = 0.001 and sulbactam: ρ = 0.74/*p* = 0.01). No significant correlation was found between plasma concentration and necrotic bone samples. The numbers of pairs were 11 for plasma and vital bone and 19 for plasma and necrotic bone.

In addition, we investigated whether the time difference between the last antibiotic administration and the collection of the bone sample affected the concentrations of ampicillin/sulbactam in bone with Spearman’s rho test. This analysis revealed a significant correlation between time difference and concentrations in vital bone for both ampicillin and sulbactam (ampicillin ρ = 0.77/*p* = 0.004 and sulbactam: ρ = 0.650/*p* = 0.022), but not in necrotic bone samples.

## 4. Discussion

We investigated the concentrations of ampicillin/sulbactam in plasma as well as vital and necrotic bone samples of patients suffering from ONJ. Our results revealed ampicillin/sulbactam concentrations in plasma of 126.3/60.2 µg/mL, which is in line with the literature values. Heibel and Foulds also investigated the ampicillin/sulbactam concentrations in plasma, finding a mean concentration (mean value from both studies) of 122.5/62.3 µg/mL [17,20]. Compared to two additional studies (97/37.6 µg/mL), the values in our study were slightly higher. This is most likely a result of the later blood sampling timepoint after infusion in that study (30 min versus 10 min in our study) because the plasma half-life of ampicillin/sulbactam is relatively short at one hour [21,22]. We can assume that all the included patients had adequate plasma antibiotic levels to reach sufficient concentrations in the jawbone.

Only a few studies have investigated the ampicillin/sulbactam concentrations in the bone to date. Most of them measured the antibiotic concentration in bone samples of healthy patients. These values were higher than the values in our study, most likely due to the compromised blood supply in ONJ patients. For example, Dehne et al. reported a mean ampicillin/sulbactam concentration of 20.7/7.7 µg/g in 40 patients [23], compared to the values of 6.3/1.9 µg/g for vital bone samples and 4.9/1.7 µg/g for necrotic bone samples, respectively, that we determined in our study. Moreover, the values in other studies revealed a similar tendency [22,24,25]. Wildfeuer et al. investigated the concentration of ampicillin/sulbactam in sternal bone and detected 17.8 µg/g for ampicillin and 8.8 µg/g for sulbactam approximately 40 min following infusion in 16 patients [22]. These concentrations were higher by factors of two to four compared to our results, which is consistent with other values in the literature [22,23,24,25].

However, we found no study investigating the concentrations in patients suffering from clinical MRONJ or ORN. Considering the pathomechanisms of MRONJ and ORN, it is certainly plausible that the concentrations of ampicillin/sulbactam in our study were lower than in healthy patients. As mentioned above, the main reason for this is most likely the compromised blood supply, which clearly limits the amount of antibiotics reaching the bone. Furthermore, this condition promotes infection and subsequent necrosis of the jawbone. Radiation therapy or antiresorptive agents probably even affect the jawbone adjacent to the necrotic areal, which may explain the low concentration also in our vital bone samples [8,26]. We found only one other study investigating the bone concentration of antibiotics in patients undergoing neoadjuvant radiation therapy of the jaw. The samples were collected approximately three weeks after radiation therapy. This study revealed significantly lower concentrations (ampicillin: 5.5 µg/mL and sulbactam: 1.2 µg/mL) than the other studies mentioned above, and clearly in line with the results of our study [17]. Patients in this study underwent radiation therapy but did not suffer from ORN.

Other studies investigating the concentration of penicillin in the jawbones of healthy patients revealed significantly lower bone concentrations than plasma concentrations [27,28]. Therefore, it is possible that the jawbone, and especially the mandible, is in a particular situation given its dense cortical structure and the blood supply through only one central vessel. Most other bones are nourished by multiple vessels penetrating the bone in various locations [29]. This could have an effect on the antibiotic bone concentration attainable through an intravenous application. Al-Nawas et al. investigated whether there is any difference between the antibiotic concentrations in the jaw and hip bones following intravenous administration of piperacillin/tazobactam in ten patients. However, this study did not reveal any significant difference in the measured concentrations [30].

We did expect the concentrations in the necrotic bone samples to be much lower than that in the vital bone because necrotic tissue does not have sufficient blood supply by definition. In support of this statement, our results reveal lower concentrations of ampicillin and sulbactam in necrotic bone. Nevertheless, this difference is very small and not significant. A possible explanation of this minor and insignificant difference is that the healthy bone and the soft tissue provide sufficient ampicillin/sulbactam concentrations, which reach the center of the necrosis by diffusion. This is supported by Spearman’s rho test, which revealed a positive correlation between the time difference between infusion of ampicillin/sulbactam and collection of the vital bone sample, but not for the necrotic bone sample collection. Furthermore, we found a significant correlation between plasma concentration and vital bone concentration but not between plasma and necrotic bone. These correlations may indicate that the vital and necrotic bone samples were taken correctly. However, a limitation of our study was that there is no clear border between vital and necrotic bone, and limited blood supply, as well as damage to the tissue architecture, is a continuum radiating from the central necrosis to the surrounding area. In an ideal world, a split-mouth model with a healthy bone sample from the contralateral side would be a better approach. However, given the medical and ethical limitations, such an approach cannot be adopted within a clinical study.

On the other hand, only a small difference in ampicillin/sulbactam concentrations between vital and necrotic bone does sound encouraging and supports the use of antibiotics in the prophylaxis and treatment of ONJ. However, as compared to the literature, the absolute concentration of ampicillin/sulbactam is rather low and the antimicrobial effect is questionable. Investigations of the microbiome in patients with ONJ revealed that *Streptococci* with a mean MIC of 0.5 µg/g (considering a plasma density of 1028 g/L [31]) cause 30–64% of infections of ONJ. Considering the current literature, the local (bone) antibiotic concentration, as measured in our study, should be sufficient to fight these bacteria [18,32,33]. The local antibiotic concentration should also be sufficient for *Escherichia coli* (MIC of 2 µg/g) [18,31]. However, the antibiotic concentration in a few patients (minimum bone ampicillin/sulbactam concentration measured was 0.4/0.3 µg/g) clearly falls below the MIC of *Escherichia coli* (MIC = 2 µg/g) and even below the MIC of *Streptococci* (MIC = 0.5 µg/g). It must be assumed that these patients would not benefit from (prolonged) antibiotic therapy.

In addition, Ewald et al. investigated bacterial colonization and antibiotic treatment in patients suffering from MRONJ. The results of the study detected a high rate of gram-negative isolates and a high rate of penicillin and ampicillin/sulbactam-resistant bacterial species. Considering that these bacteria grow in biofilms, which means higher MICs, the observed antibiotic bone concentrations may not be sufficient [34,35,36]. We have previously discussed the positive effect of PRF given its antibiotic load to increase the local antibiotic concentrations in the treatment of patients suffering from ONJ. We further demonstrated that the application of PRF unfolds high local antibiotic concentrations, which have an antimicrobial effect. In view of the discussed issues, this could be particularly beneficial [16,18,33].

A limitation of our study is the small study collective with only 13 vital and 21 necrotic bone samples. This fact makes subgroup analyses impossible, for example, a comparison of ORN and MRONJ. Furthermore, any comparison between bone concentrations in the upper and lower jaw could not be performed. Our study did not differentiate the various stages of MRONJ and ORN, but it is conceivable that antibiotic concentrations may differ depending on the stage of MRONJ. Another limitation is that obtaining bone samples proved difficult in some cases. While necrotic bone can be easily located, assessment of the vital areas is only possible through clinical evaluation (for example visual or bleeding of the bone). This depends on the surgeon on the one hand, but also on clinical circumstances (not enlarging the surgical area unnecessarily to obtain a vital specimen). Histologic assessment of whether a vital bone is present or not was not possible in this study setting. It is worth noting that the correlation between the last antibiotic treatment and concentration in vital but not necrotic bone indirectly indicates a difference in the samples taken (vital versus necrotic). Finally, we believe this is the first study to investigate the antibiotic concentration in both the necrotic and vital bone of ONJ patients, and our results provide valuable information with respect to the purpose and benefit of antibiotic therapy in these patients.

## 5. Conclusions

Summarizing the results of our study, intravenous antibiotic therapy with ampicillin/sulbactam seems capable of reaching clinically sufficient bone concentrations. We detected no significant difference in ampicillin/sulbactam concentrations when comparing healthy and necrotic bone tissue. It is important to note that the concentration in the bone samples is up to a factor of 20 times lower than the plasma concentration, indicating that jaw bone is strikingly different from plasma as a compartment. Nevertheless, it should be taken into account that antibiotic uptake by bone probably occurs with a time delay. We may hypothesize that when therapy is applied orally (for example, with amoxicillin/clavulanic acid, 875/125 mg twice a day), which results in much lower concentrations than through intravenous application, the antibiotic concentration in bone may fall below the respective MICs of *Escherichia coli*, *Streptococci*, as well as other species of bacteria. Further prospective studies with a larger sample size are necessary to clarify these concerns and especially the benefit of antibiotic therapy in patients suffering from ONJ.

## Figures and Tables

**Table 1:** Patient characteristics.

	Participants
N (total)	21
Plasma samples	19
Vital bone samples	13
Necrotic bone samples	21
m/f	11/10
Mean age (in years)	69 (SD ± 8.9)
Min and max age	55–85
ORN	5
MRONJ	16
Localization	
Upper jaw	3
Lower jaw	18
Renal function (MD ± SD):	73.1 ± 24.4 mL/min *

N: number of participants, m: male, f: female, ORN: osteoradionecrosis, MRONJ: antiresorptive-agent-related osteonecrosis of the jaw. * Glomerular filtration rate (MDRD) in mL/min.

**Table 2:** Concentrations of ampicillin and sulbactam in plasma.

	Ampicillin	Sulbactam
N	19	19
Concentration *	126.3	60.2
SD	±77.6	±35.0
95% CI *	88.9–163.8	43.3–77.1
Minimum *	2.6	2.1
Maximum *	262.9	120.6

N: number of patients; SD: standard deviation; 95% CI: 95% confidence interval. * Concentrations were in µg/mL.

**Table 3:** Concentrations of ampicillin and sulbactam in vital bone samples.

	Ampicillin	Sulbactam
N	13	13
Concentration *	6.3	1.9
SD	±7.8	±2.0
95% CI *	1.6–11.0	0.7–3.1
Minimum *	0.4	0.3
Maximum *	27.1	6.8
Median	2.7	1.4

N: number of patients; SD: standard deviation; 95% CI: 95% confidence interval. * Concentrations were in µg/g.

**Table 4:** Concentrations of ampicillin and sulbactam in necrotic bone samples.

	Ampicillin	Sulbactam
N	21	21
Concentration *	4.9	1.7
SD	±7.0	±1.7
95% CI *	1.7–8.1	0.9–2.5
Minimum *	0.6	0.3
Maximum *	32.5	7.6
Median	2.5	1.3

N: number of patients; SD: standard deviation; 95% CI: 95% confidence interval. * Concentrations were in µg/g.

## Data Availability

The dataset used to reach the conclusions in this article is included within the article. Further clinical data and information are not publicly available because other, currently unpublished, studies are based on it. However, these are available from the corresponding author upon reasonable request.

## Supplementary Materials

The following supporting information can be downloaded at: https://www.mdpi.com/article/10.3390/ijerph192214917/s1, Table S1: Table showing the mean concentration of ampicillin and sulbactam separately for the upper and lower jaw; Table S2: Table showing the mean concentration of ampicillin and sulbactam with respect to the etiology of the osteonecrosis.
