# Supplementary material for: Bone Concentration of Ampicillin/Sulbactam: A Pilot Study in Patients with Osteonecrosis of the Jaw
Source: Int J Environ Res Public Health. 2022 Nov 13;19(22):14917. doi: 10.3390/ijerph192214917 (PMC9690242; doi:10.3390/ijerph192214917)
Supplement: Supplementary file 1 [file ijerph-19-14917-s001.zip › ijerph-2010670-supplementary.pdf]

Supplement

Table S1: Table showing the mean concentration of ampicillin and sulbactam separately for the upper and lower jaw.

	**Upper jaw**		**Lower jaw**	
	**Ampicillin**	**Sulbactam**	**Ampicillin**	**Sulbactam**
N	3	3	18	18
Concentration *	15.3/32.5	3.56/7.6	5.6/3.56	1.8/1.4

* concentration vital/necrotic bone

Table S2: Table showing the mean concentration of ampicillin and sulbactam with respect to the etiology of the osteonecrosis.

	**MRONJ**		**ONJ**	
	**Ampicillin**	**Sulbactam**	**Ampicillin**	**Sulbactam**
N	16	16	5	5
Concentration *	5.7/3.6	1.5/1.5	8.3/10.4	3.4/2.8

* concentration vital/necrotic bone
